# Crystal structure of methyl (*Z*)-2-[(*Z*)-3-methyl-2-({(*E*)-1-[(R*)-4-methyl­cyclo­hex-3-en-1-yl]ethyl­idene}hydrazinyl­idene)-4-oxo­thia­zolidin-5-yl­idene]acetate

**DOI:** 10.1107/S2056989017014311

**Published:** 2017-10-13

**Authors:** Mourad Fawzi, Aziz Auhmani, Moulay Youssef Ait Itto, Abdelkhalek Riahi, Sylviane Chevreux, El Mostafa Ketatni

**Affiliations:** a Laboratory of Organic Synthesis and Physico-Molecular Chemistry, Department of Chemistry, Faculty of Sciences Semlalia, PO Box 2390, Marrakech 40001, Morocco; b Institute of Molecular Chemistry of Reims, CNRS UMR 7312 Bat. Europol Agro, Moulin of the Housse UFR Sciences, PO Box 1039-51687 Reims Cedex 2, France; c Laboratory of Applied Spectro-Chemistry and the Environment, University Sultan Moulay Slimane, Faculty of Sciences and Technology, PO Box 523, Beni-Mellal, Morocco

**Keywords:** crystal structure: hydrazine: thia­zol­idinone, C—H⋯O hydrogen bonds, offset π–π inter­actions

## Abstract

The new title 4-thia­zolidinone derivative, C_16_H_21_N_3_O_3_S, was obtained from the cyclization reaction of 4-methyl-3-thio­semicarbazone and dimethyl acetyl­enedi­carboxyl­ate (DMAD). The cyclo­hexyl­idene ring has an envelope conformation with the stereogenic centre C atom as the flap. Its mean plane makes a dihedral angle of 56.23 (9)° with the thia­zolidine ring mean plane. In the crystal, mol­ecules are linked by C—H⋯O hydrogen bonds forming chains propagating in the [001] direction. Within the chains there are offset π–π inter­actions between the thia­zolidine rings of inversion-related mol­ecules [centroid–centroid distance = 3.703 (1) Å]. The chains are linked by further C—H⋯O hydrogen bonds, forming slabs parallel to the *ac* plane.

## Abstract 2

A new 4-thia­zolidinone derivative has been obtained from the cyclization reaction of 4-methyl-3-thio­semicarbazone and dimethyl acetyl­enedi­carboxyl­ate (DMAD).

## Chemical context   

It has been reported that thia­zolidinones exhibit anti­bacterial (Mayekar & Mulwad, 2008 ▸), anti­fungal (Omar *et al.* 2010 ▸), anti­convulsant (Bhat *et al.*, 2008 ▸), anti­tubercular (Babaoglu *et al.*, 2003 ▸), anti-inflammatory (Vigorita *et al.* 2003 ▸), anti­histaminic (Agrawal *et al.*, 2000 ▸), cardiovascular (Suzuki *et al.*, 1999 ▸) and anti-HIV (Rawal *et al.*, 2005 ▸) activities.

**Figure 1:**
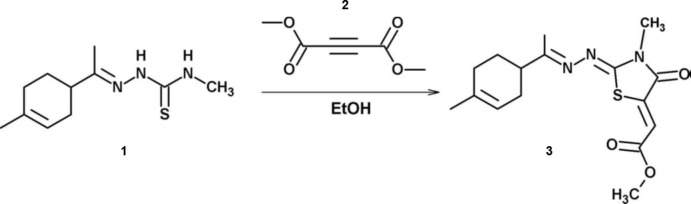
Reaction scheme for the synthesis of title compound **3**.

With the aim of preparing new thia­zolidinone derivatives, we report herein on the synthesis (Fig. 1 ▸) and crystal structure of the title compound **3**, from 4-methyl-3-thio­semicarbazone **1**. Treatment of **1** with dimethyl acetyl­enedi­carboxyl­ate **2** in boiling ethanol for 1 h, afforded the thia­zolidin-4-one **3** in 90% yield. Its structure has also been fully characterized by NMR spectroscopy while its relative stereochemistry was determined based mainly on the synthetic pathway and implied by the X-ray diffraction analysis.
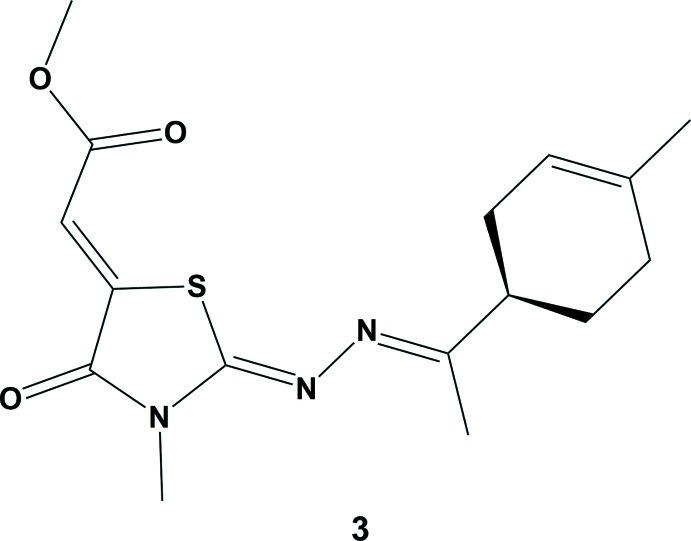


## Structural commentary   

**Figure 2:**
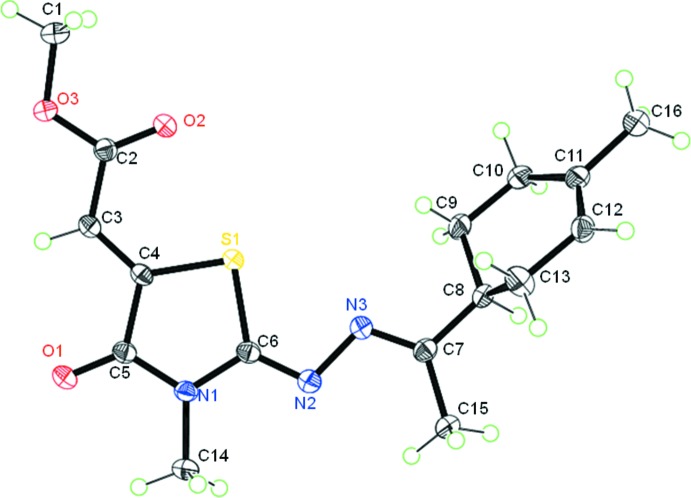
The mol­ecular structure of the title compound **3**, with the atom labelling. Displacement ellipsoids are drawn at the 30% probability level.

The title compound **3**, is built up from an thia­zolidinone ring linked to cyclo­hexyl­idene-hydrazone and meth­oxy-oxo­ethyl­idene units (Fig. 2 ▸). The compound crystallizes in the centrosymmetric space group *P* 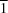, and the stereogenic centre at C8 was assigned as having an *R* configuration. As expected, the thia­zolidine ring and all the atoms attached to it (plane *A* = S1/C4/C5/N1/C6/N2/N3/O1/C3/C14) are roughly coplanar with an r.m.s. deviation of 0.036 Å. Its mean plane makes a dihedral angle of 56.0 (1)° with the mean plane of the cyclo­hexyl­idene ring (C8-C13). The meth­oxy­carbonyl group (C1/O2/O3/C2) is also twisted slightly with respect to plane *A*, their mean planes being inclined to one another by 11.2 (2)°. The six-membered cyclo­hexyl­idene ring has an envelope conformation with atom C8 as the flap: puckering parameters are *Q* = 0.494 (2) Å, θ = 129.8 (2)° and φ = 180.8 (3)°. The C7=N3 and N2=C6 bond lengths are 1.282 (2) and 1.278 (2) Å, respectively, consistent with C=N double bonding. The C6—N2—N3—C7, C4—C3—C2—O3 and C3—C2—O3—C1 torsion angles are 175.5 (2), −172.4 (2) and 172.5 (2)°, respectively.

## Supra­molecular features   

**Figure 3:**
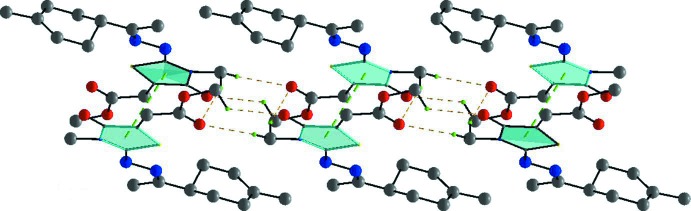
Partial crystal packing for title compound **3**, showing the C3—H3⋯O3^i^ hydrogen bonds and the offset π–π inter­actions between inversion-related mol­ecules, forming chains in the [001] direction (dashed lines; only atom H3 has been included).

**Figure 4:**
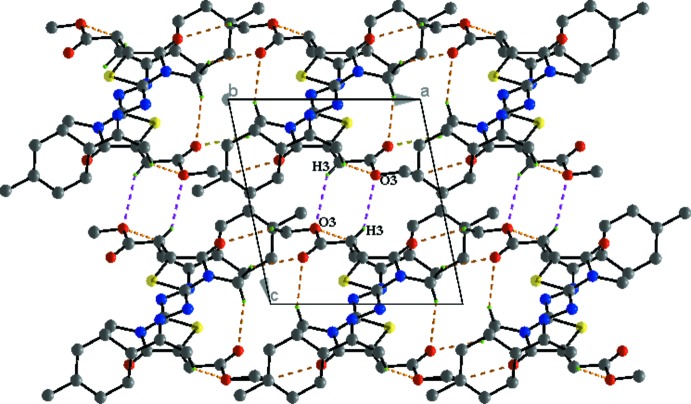
Packing and hydrogen-bonding inter­actions of the title compound viewed along the *b* axis. For clarity, only the H atoms involved in the hydrogen bonds (dashed lines) inter­actions have been included.

In the crystal, mol­ecules are linked C3—H3⋯O3^i^ hydrogen bonds, forming chains propagating along [001]; see Table 1 ▸ and Fig. 3 ▸. Within the chains there are weak offset π–π stacking inter­actions between inversion-related thia­zole rings [see Fig. 3 ▸; *Cg*1⋯*Cg*1(−*x* + 1, −*y* + 1, −*z*) = 3.703 (1) Å,where *Cg*1 is the centroid of the S1/N1/C4–C6 ring, inter­planar distance = 3.468 (1) Å, slippage = 1.298 Å]. The chains are linked by further C—H⋯O hydrogen bonds, forming slabs lying parallel to the *ac* plane (Table 1 ▸, Figs. 4 ▸ and 5 ▸).

## Database survey   

A search of the Cambridge Structural Database (CSD, Version 5.38, last update May 2017; Groom *et al.*, 2016 ▸) using a thia­zolidone substituted by meth­oxy-oxo­ethyll­idene and methyl­hydrazone as the main skeleton gave eight hits. The most relevant structures are methyl (2-{[1-(4-hy­droxy­phen­yl)ethyl­idene]hydrazono}-4-oxo-3-phenyl-1,3-thia­zolidin-5-yl­idene)acetate (AGOMUG; Mohamed, Mague *et al.*, 2013 ▸), methyl (2-{[1-(4-methyl­phen­yl)ethyl­idene]hydrazono}-4-oxo-3-phenyl-1,3-thia­zolidin-5-yl­idene)acetate (NIPPAF; Mague *et al.*, 2013 ▸) and dimethyl 2-[(4-{*N*-[5-(2-meth­oxy-2-oxo­ethyl­idene)-4-oxo-3-phenyl-1,3-thia­zolidin-2-yl­idene]ethane­hydrazono­yl}phen­yl)amino]­but-2-enedioate (RIMDIC; Mohamed, Akkurt *et al.*, 2013 ▸).

A comparison of the main C—N, N—N, C—S bond lengths in the title compound and the structures extracted from the CSD shows a good correlation. The C=N—N=C torsion angles indicate that in each case the four atoms are nearly planar, *viz*. 175.5 (2)° in the title compound, 172.1 (2)° in AGOMUG, −178.9 (2) and −165.5 (2)° in NIPPAF and −167.4 (5)° in RIMDIC.

## Synthesis and crystallization   

To a solution of 4-methyl-3-thio­semicarbazone (200 mg, 1.33 mmol) in ethanol (15 ml) was added dimethyl acetyl­enedi­carboxyl­ate (DMAD) (0.24 ml, 1.66 mmol). The mixture was stirred under reflux for 1 h, leading to the corresponding thia­zolidinone. After cooling, the mixture was extracted with ethyl acetate (3 × 20 ml). The organic layer was washed with water, dried on anhydrous Na_2_SO_4_ and then evaporated under reduced pressure. The obtained residue was chromatographed on a silica gel column using hexane as eluent, to give compound **3** (yield 404 mg, 90%). Yellow prismatic crystals were obtained from a petroleum ether solution, by slow evaporation of the solvent at room temperature.

## Refinement   

Crystal data, data collection and structure refinement details are summarized in Table 2 ▸. The C-bound H atoms were placed in calculated positions with C—H = 0.95–1.00 Å, and refined in the riding-model approximation with *U*_iso_(H) = 1.5*U*_eq_(C-meth­yl) and 1.2*U*_eq_(C) for other H atoms.

## Supplementary Material

Crystal structure: contains datablock(s) I, Global. DOI: 10.1107/S2056989017014311/su5396sup1.cif

Structure factors: contains datablock(s) I. DOI: 10.1107/S2056989017014311/su5396Isup2.hkl

Supporting information file. DOI: 10.1107/S2056989017014311/su5396Isup3.cml

CCDC reference: 1577993

Additional supporting information:  crystallographic information; 3D view; checkCIF report

## Figures and Tables

**Figure 5:**
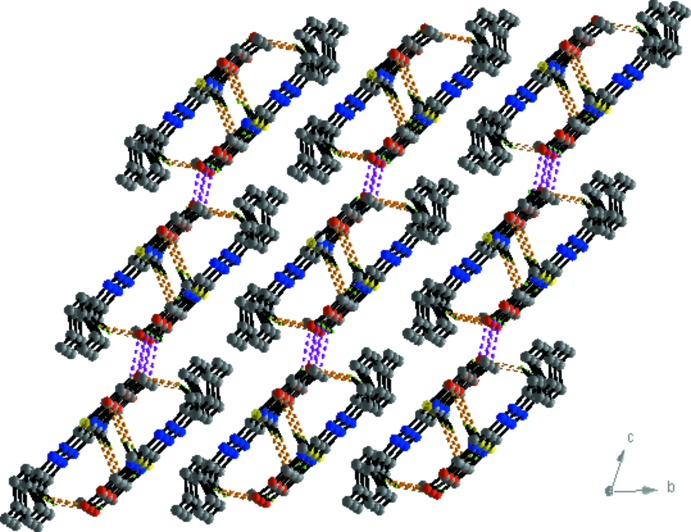
Packing and hydrogen-bonding inter­actions of the title compound, viewed along the *a* axis. For clarity, only the H atoms involved in hydrogen bonding (dashed lines) have been included.

**Table 1:** Hydrogen-bond geometry (Å, °)

*D*—H⋯*A*	*D*—H	H⋯*A*	*D*⋯*A*	*D*—H⋯*A*
C3—H3⋯O3^i^	0.95	2.59	3.4133 (19)	146
C1—H1*C*⋯O1^ii^	0.98	2.51	3.208 (2)	128
C14—H14*A*⋯O2^iii^	0.98	2.45	3.252 (2)	139
C14—H14*B*⋯O2^iv^	0.98	2.48	3.409 (2)	159
C15—H15*A*⋯O3^iv^	0.98	2.55	3.351 (2)	139

Symmetry codes: (i) 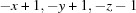; (ii) 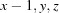; (iii) 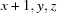; (iv) 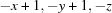.

**Table 2:** Experimental details

Crystal data	
Chemical formula	C_16_H_21_N_3_O_3_S
*M*_r_	335.42
Crystal system, space group	Triclinic, *P* 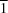
Temperature (K)	100
*a*, *b*, *c* (Å)	9.0982 (2), 9.9556 (3), 10.5071 (3)
α, β, γ (°)	66.772 (1), 74.572 (1), 77.706 (1)
*V* (Å^3^)	836.76 (4)
*Z*	2
Radiation type	Cu *K*α
μ (mm^−1^)	1.88
Crystal size (mm)	0.39 × 0.28 × 0.20

Data collection	
Diffractometer	D8 Venture CMOS area detector
Absorption correction	Numerical (*SADABS*; Bruker, 2012 ▸)
*T*_min_, *T*_max_	0.733, 0.919
No. of measured, independent and observed [*I* > 2σ(*I*)] reflections	30681, 3405, 3195
*R*_int_	0.035
(sin θ/λ)_max_ (Å^−1^)	0.625

Refinement	
*R*[*F* ^2^ > 2σ(*F* ^2^)], *wR*(*F* ^2^), *S*	0.038, 0.102, 1.06
No. of reflections	3405
No. of parameters	212
H-atom treatment	H-atom parameters constrained
Δρ_max_, Δρ_min_ (e Å^−3^)	0.42, −0.46

Computer programs: *APEX2* and *SAINT* (Bruker, 2012 ▸), *SHELXS2014* (Sheldrick, 2008 ▸), *SHELXL2014* (Sheldrick, 2015 ▸), *ORTEP-3 for Windows* (Farrugia, 2012 ▸), *DIAMOND* (Brandenburg & Putz, 2012 ▸), *PLATON* (Spek, 2009 ▸) and *publCIF* (Westrip, 2010 ▸).

## supplementary crystallographic information

### Crystal data

**Table:**

C_16_H_21_N_3_O_3_S	*Z* = 2
*M**_r_* = 335.42	*F*(000) = 356
Triclinic, *P*1	*D*_x_ = 1.331 Mg m^−^^3^
*a* = 9.0982 (2) Å	Cu *K*α radiation, λ = 1.54178 Å
*b* = 9.9556 (3) Å	Cell parameters from 9903 reflections
*c* = 10.5071 (3) Å	θ = 4.9–74.5°
α = 66.772 (1)°	µ = 1.88 mm^−^^1^
β = 74.572 (1)°	*T* = 100 K
γ = 77.706 (1)°	Prismatic, yellow
*V* = 836.76 (4) Å^3^	0.39 × 0.28 × 0.20 mm

### Data collection

**Table:**

D8 Venture CMOS area detector diffractometer	3195 reflections with *I* > 2σ(*I*)
Radiation source: microsource	*R*_int_ = 0.035
φ and ω scans	θ_max_ = 74.5°, θ_min_ = 4.7°
Absorption correction: numerical (SADABS; Bruker, 2012)	*h* = −11→11
*T*_min_ = 0.733, *T*_max_ = 0.919	*k* = −12→12
30681 measured reflections	*l* = −12→13
3405 independent reflections	

### Refinement

**Table:**

Refinement on *F*^2^	Primary atom site location: structure-invariant direct methods
Least-squares matrix: full	Secondary atom site location: difference Fourier map
*R*[*F*^2^ > 2σ(*F*^2^)] = 0.038	Hydrogen site location: inferred from neighbouring sites
*wR*(*F*^2^) = 0.102	H-atom parameters constrained
*S* = 1.06	*w* = 1/[σ^2^(*F*_o_^2^) + (0.048*P*)^2^ + 0.6482*P*] where *P* = (*F*_o_^2^ + 2*F*_c_^2^)/3
3405 reflections	(Δ/σ)_max_ = 0.016
212 parameters	Δρ_max_ = 0.42 e Å^−^^3^
0 restraints	Δρ_min_ = −0.46 e Å^−^^3^

### Special details

**Table:**

Geometry. All esds (except the esd in the dihedral angle between two l.s. planes) are estimated using the full covariance matrix. The cell esds are taken into account individually in the estimation of esds in distances, angles and torsion angles; correlations between esds in cell parameters are only used when they are defined by crystal symmetry. An approximate (isotropic) treatment of cell esds is used for estimating esds involving l.s. planes.

### Fractional atomic coordinates and isotropic or equivalent isotropic displacement parameters (Å^2^)

**Table:**

	*x*	*y*	*z*	*U*_iso_*/*U*_eq_	
S1	0.40715 (4)	0.68958 (4)	−0.11410 (4)	0.01854 (12)	
O1	0.80122 (13)	0.54559 (13)	−0.29654 (13)	0.0255 (3)	
O2	0.21903 (13)	0.59108 (14)	−0.22869 (13)	0.0262 (3)	
O3	0.31331 (13)	0.45665 (12)	−0.36926 (12)	0.0219 (3)	
N1	0.70665 (15)	0.66298 (14)	−0.13721 (14)	0.0192 (3)	
N2	0.56362 (16)	0.77397 (15)	0.02334 (15)	0.0222 (3)	
N3	0.40773 (15)	0.81672 (15)	0.07586 (15)	0.0215 (3)	
C1	0.15960 (19)	0.4245 (2)	−0.3520 (2)	0.0267 (4)	
H1A	0.1267	0.3563	−0.2554	0.040*	
H1B	0.1594	0.3793	−0.4196	0.040*	
H1C	0.0887	0.5160	−0.3692	0.040*	
C2	0.32641 (18)	0.53614 (17)	−0.29626 (17)	0.0194 (3)	
C3	0.48814 (18)	0.54559 (17)	−0.30630 (17)	0.0205 (3)	
H3	0.5657	0.5084	−0.3690	0.025*	
C4	0.52821 (17)	0.60574 (17)	−0.22856 (17)	0.0184 (3)	
C5	0.69402 (18)	0.59946 (17)	−0.22780 (17)	0.0193 (3)	
C6	0.56930 (18)	0.71493 (17)	−0.06627 (17)	0.0189 (3)	
C7	0.38679 (18)	0.86854 (17)	0.17420 (17)	0.0201 (3)	
C8	0.22330 (18)	0.90988 (17)	0.23980 (17)	0.0200 (3)	
H8	0.2202	1.0019	0.2579	0.024*	
C9	0.1056 (2)	0.9394 (2)	0.14898 (19)	0.0281 (4)	
H9A	0.1337	1.0203	0.0579	0.034*	
H9B	0.1069	0.8502	0.1285	0.034*	
C10	−0.0542 (2)	0.9808 (2)	0.22322 (18)	0.0263 (4)	
H10	−0.1269	1.0467	0.1702	0.032*	
C11	−0.0941 (2)	0.92015 (19)	0.3727 (2)	0.0259 (4)	
C12	0.01418 (19)	0.83015 (18)	0.45599 (18)	0.0235 (3)	
H12A	−0.0295	0.7375	0.5188	0.028*	
H12B	0.0236	0.8805	0.5175	0.028*	
C13	0.1742 (2)	0.7892 (2)	0.38279 (19)	0.0289 (4)	
H13A	0.1778	0.6962	0.3681	0.035*	
H13B	0.2474	0.7725	0.4440	0.035*	
C14	0.85481 (18)	0.66081 (19)	−0.10627 (19)	0.0243 (4)	
H14A	0.9379	0.6357	−0.1775	0.036*	
H14B	0.8625	0.5871	−0.0124	0.036*	
H14C	0.8633	0.7581	−0.1082	0.036*	
C15	0.51144 (19)	0.88538 (19)	0.23429 (18)	0.0239 (3)	
H15A	0.5290	0.7966	0.3162	0.036*	
H15B	0.4803	0.9709	0.2638	0.036*	
H15C	0.6064	0.8996	0.1621	0.036*	
C16	−0.2564 (2)	0.9523 (2)	0.4458 (2)	0.0317 (4)	
H16A	−0.3170	0.8763	0.4573	0.048*	
H16B	−0.3016	1.0488	0.3888	0.048*	
H16C	−0.2564	0.9526	0.5390	0.048*	

### Atomic displacement parameters (Å^2^)

**Table:**

	*U*^11^	*U*^22^	*U*^33^	*U*^12^	*U*^13^	*U*^23^
S1	0.01467 (19)	0.0227 (2)	0.0196 (2)	−0.00267 (14)	−0.00178 (14)	−0.00991 (15)
O1	0.0160 (5)	0.0333 (6)	0.0293 (7)	−0.0041 (5)	0.0009 (5)	−0.0163 (5)
O2	0.0173 (6)	0.0360 (7)	0.0308 (7)	−0.0030 (5)	−0.0017 (5)	−0.0198 (5)
O3	0.0184 (5)	0.0264 (6)	0.0251 (6)	−0.0044 (4)	−0.0042 (4)	−0.0130 (5)
N1	0.0152 (6)	0.0218 (6)	0.0207 (7)	−0.0042 (5)	−0.0028 (5)	−0.0073 (5)
N2	0.0195 (7)	0.0258 (7)	0.0221 (7)	−0.0038 (5)	−0.0033 (5)	−0.0097 (6)
N3	0.0195 (7)	0.0250 (7)	0.0217 (7)	−0.0035 (5)	−0.0025 (5)	−0.0110 (6)
C1	0.0207 (8)	0.0324 (9)	0.0324 (10)	−0.0078 (7)	−0.0060 (7)	−0.0144 (8)
C2	0.0196 (8)	0.0206 (7)	0.0179 (8)	−0.0039 (6)	−0.0031 (6)	−0.0063 (6)
C3	0.0170 (7)	0.0240 (8)	0.0206 (8)	−0.0040 (6)	−0.0001 (6)	−0.0097 (6)
C4	0.0149 (7)	0.0197 (7)	0.0185 (8)	−0.0035 (6)	−0.0002 (6)	−0.0060 (6)
C5	0.0167 (7)	0.0206 (7)	0.0191 (8)	−0.0048 (6)	−0.0011 (6)	−0.0057 (6)
C6	0.0172 (7)	0.0190 (7)	0.0187 (8)	−0.0036 (6)	−0.0030 (6)	−0.0045 (6)
C7	0.0228 (8)	0.0196 (7)	0.0182 (8)	−0.0043 (6)	−0.0052 (6)	−0.0055 (6)
C8	0.0224 (8)	0.0216 (7)	0.0184 (8)	−0.0029 (6)	−0.0050 (6)	−0.0092 (6)
C9	0.0258 (9)	0.0407 (10)	0.0236 (9)	0.0015 (7)	−0.0088 (7)	−0.0180 (8)
C10	0.0229 (8)	0.0360 (9)	0.0225 (9)	−0.0026 (7)	−0.0083 (7)	−0.0111 (7)
C11	0.0227 (8)	0.0282 (8)	0.0349 (10)	−0.0060 (7)	−0.0035 (7)	−0.0197 (8)
C12	0.0267 (8)	0.0240 (8)	0.0210 (8)	−0.0058 (6)	−0.0024 (6)	−0.0096 (7)
C13	0.0260 (9)	0.0283 (9)	0.0239 (9)	0.0004 (7)	−0.0038 (7)	−0.0032 (7)
C14	0.0165 (8)	0.0305 (9)	0.0277 (9)	−0.0028 (6)	−0.0062 (6)	−0.0114 (7)
C15	0.0232 (8)	0.0284 (8)	0.0247 (8)	−0.0027 (6)	−0.0074 (7)	−0.0130 (7)
C16	0.0241 (9)	0.0427 (10)	0.0343 (10)	−0.0031 (7)	−0.0046 (7)	−0.0215 (9)

### Geometric parameters (Å, º)

**Table:**

S1—C4	1.7469 (16)	C8—H8	1.0000
S1—C6	1.7736 (16)	C9—C10	1.512 (2)
O1—C5	1.212 (2)	C9—H9A	0.9900
O2—C2	1.211 (2)	C9—H9B	0.9900
O3—C2	1.3403 (19)	C10—C11	1.416 (3)
O3—C1	1.4495 (19)	C10—H10	0.9500
N1—C5	1.372 (2)	C11—C12	1.415 (2)
N1—C6	1.385 (2)	C11—C16	1.504 (2)
N1—C14	1.462 (2)	C12—C13	1.509 (2)
N2—C6	1.278 (2)	C12—H12A	0.9900
N2—N3	1.4174 (19)	C12—H12B	0.9900
N3—C7	1.282 (2)	C13—H13A	0.9900
C1—H1A	0.9800	C13—H13B	0.9900
C1—H1B	0.9800	C14—H14A	0.9800
C1—H1C	0.9800	C14—H14B	0.9800
C2—C3	1.467 (2)	C14—H14C	0.9800
C3—C4	1.340 (2)	C15—H15A	0.9800
C3—H3	0.9500	C15—H15B	0.9800
C4—C5	1.499 (2)	C15—H15C	0.9800
C7—C15	1.503 (2)	C16—H16A	0.9800
C7—C8	1.508 (2)	C16—H16B	0.9800
C8—C9	1.527 (2)	C16—H16C	0.9800
C8—C13	1.532 (2)		
			
C4—S1—C6	90.05 (7)	C10—C9—H9B	109.4
C2—O3—C1	115.77 (13)	C8—C9—H9B	109.4
C5—N1—C6	115.69 (13)	H9A—C9—H9B	108.0
C5—N1—C14	121.75 (13)	C11—C10—C9	119.25 (15)
C6—N1—C14	122.23 (13)	C11—C10—H10	120.4
C6—N2—N3	108.81 (13)	C9—C10—H10	120.4
C7—N3—N2	114.54 (13)	C12—C11—C10	122.07 (16)
O3—C1—H1A	109.5	C12—C11—C16	118.75 (16)
O3—C1—H1B	109.5	C10—C11—C16	119.18 (16)
H1A—C1—H1B	109.5	C11—C12—C13	118.89 (15)
O3—C1—H1C	109.5	C11—C12—H12A	107.6
H1A—C1—H1C	109.5	C13—C12—H12A	107.6
H1B—C1—H1C	109.5	C11—C12—H12B	107.6
O2—C2—O3	124.53 (14)	C13—C12—H12B	107.6
O2—C2—C3	124.24 (15)	H12A—C12—H12B	107.0
O3—C2—C3	111.22 (13)	C12—C13—C8	111.68 (14)
C4—C3—C2	121.12 (14)	C12—C13—H13A	109.3
C4—C3—H3	119.4	C8—C13—H13A	109.3
C2—C3—H3	119.4	C12—C13—H13B	109.3
C3—C4—C5	120.42 (14)	C8—C13—H13B	109.3
C3—C4—S1	127.81 (12)	H13A—C13—H13B	107.9
C5—C4—S1	111.70 (11)	N1—C14—H14A	109.5
O1—C5—N1	124.96 (14)	N1—C14—H14B	109.5
O1—C5—C4	125.02 (15)	H14A—C14—H14B	109.5
N1—C5—C4	110.01 (13)	N1—C14—H14C	109.5
N2—C6—N1	122.47 (14)	H14A—C14—H14C	109.5
N2—C6—S1	125.00 (12)	H14B—C14—H14C	109.5
N1—C6—S1	112.53 (11)	C7—C15—H15A	109.5
N3—C7—C15	125.43 (15)	C7—C15—H15B	109.5
N3—C7—C8	117.47 (14)	H15A—C15—H15B	109.5
C15—C7—C8	117.04 (14)	C7—C15—H15C	109.5
C7—C8—C9	115.06 (13)	H15A—C15—H15C	109.5
C7—C8—C13	109.94 (13)	H15B—C15—H15C	109.5
C9—C8—C13	108.72 (14)	C11—C16—H16A	109.5
C7—C8—H8	107.6	C11—C16—H16B	109.5
C9—C8—H8	107.6	H16A—C16—H16B	109.5
C13—C8—H8	107.6	C11—C16—H16C	109.5
C10—C9—C8	111.08 (14)	H16A—C16—H16C	109.5
C10—C9—H9A	109.4	H16B—C16—H16C	109.5
C8—C9—H9A	109.4		

### Hydrogen-bond geometry (Å, º)

**Table:**

*D*—H···*A*	*D*—H	H···*A*	*D*···*A*	*D*—H···*A*
C3—H3···O3^i^	0.95	2.59	3.4133 (19)	146
C1—H1*C*···O1^ii^	0.98	2.51	3.208 (2)	128
C14—H14*A*···O2^iii^	0.98	2.45	3.252 (2)	139
C14—H14*B*···O2^iv^	0.98	2.48	3.409 (2)	159
C15—H15*A*···O3^iv^	0.98	2.55	3.351 (2)	139

Symmetry codes: (i) −*x*+1, −*y*+1, −*z*−1; (ii) *x*−1, *y*, *z*; (iii) *x*+1, *y*, *z*; (iv) −*x*+1, −*y*+1, −*z*.
